# Mild Propofol Sedation Reduces Frontal Lobe and Thalamic Cerebral Blood Flow: An Arterial Spin Labeling Study

**DOI:** 10.3389/fphys.2019.01541

**Published:** 2019-12-18

**Authors:** Neeraj Saxena, Tommaso Gili, Ana Diukova, Danielle Huckle, Judith E. Hall, Richard G. Wise

**Affiliations:** ^1^Cardiff University Brain Research Imaging Centre (CUBRIC), School of Psychology, Cardiff University, Cardiff, United Kingdom; ^2^Department of Anaesthetics, Intensive Care and Pain Medicine, Cwm Taf Morgannwg University Health Board, Llantrisant, United Kingdom; ^3^IMT School for Advanced Studies Lucca, Lucca, Italy; ^4^Department of Anaesthetics, University Hospital of Wales, Cardiff, United Kingdom; ^5^Department of Anaesthetics, Intensive Care and Pain Medicine, School of Medicine, Cardiff University, Cardiff, United Kingdom

**Keywords:** arterial spin labeling, functional magnetic resonance imaging, cerebral blood flow, propofol, sedation

## Abstract

Mechanisms of anesthetic drug-induced sedation and unconsciousness are still incompletely understood. Functional neuroimaging modalities provide a window to study brain function changes during anesthesia allowing us to explore the sequence of neuro-physiological changes associated with anesthesia. Cerebral perfusion change under an assumption of intact neurovascular coupling is an indicator of change in large-scale neural activity. In this experiment, we have investigated resting state cerebral blood flow (CBF) changes in the human brain during mild sedation, with propofol. Arterial spin labeling (ASL) provides a non-invasive, reliable, and robust means of measuring cerebral blood flow (CBF) and can therefore be used to investigate central drug effects. Mild propofol sedation-related CBF changes were studied at rest (*n* = 15), in a 3 T MR scanner using a PICORE-QUIPSS II ASL technique. CBF was reduced in bilateral paracingulate cortex, premotor cortex, Broca’s areas, right superior frontal gyrus and also the thalamus. This cerebral perfusion study demonstrates that propofol induces suppression of key cortical (frontal lobe) and subcortical (thalamus) regions during mild sedation.

## Introduction

Mechanisms of anesthetic drug-induced sedation and unconsciousness are still incompletely understood. While neuroimaging studies suggest a reduction of activity in a number of cortical and subcortical areas along with breakdown of functional connectivity of thalamo-cortical, frontoparietal, or default mode networks (DMNs), the sequence and specificity of these changes remain disputed (Lee et al., 2009; Boveroux et al., 2010). Apart from revealing systems-level mechanisms of anesthesia, understanding brain perfusion and its alterations with sedation/anesthesia is thought to be helpful in exploiting the neuroprotective effects of these drugs in brain-injured (traumatic or stroke) patients receiving sedation or those undergoing neuro-anesthesia.

Techniques including Positron Emission Tomography and Blood Oxygen Level Dependent (BOLD) contrast-based Functional magnetic resonance imaging (fMRI) have been utilized to explore the neural correlates of anesthetic-related changes in consciousness and arousal. Recent improvements in arterial spin labeling (ASL) methodology, a measurement of tissue blood flow, render it practical to quantify pharmacological effects in the human brain. It is completely non-invasive, being based on an endogenous tracer (magnetically labeled arterial blood) and, since the perfusion signal is encoded in the difference between control and tagged images, it is minimally affected by baseline drift, making it suitable for long-term studies or those with low frequency changes. ASL-based techniques are, therefore, especially suited for physiological and pharmacological studies of brain activity.

Propofol, a GABA-ergic agonist compound, is an anesthetic drug widely used for sedation and anesthetic induction and maintenance. Recently, Qiu et al. (2017) have shown perfusion changes with deep sedation (a state characterized by unresponsiveness to verbal commands) induced with propofol, in the frontoparietal, DMN, visual networks, and thalamus.

In this experiment, we have used ASL to investigate resting state cerebral blood flow (CBF) changes in the human brain during mild sedation. We chose “mild” sedation as the earliest, objectively defined, step change in consciousness to evaluate the earliest changes in neural mechanisms associated with altered arousal. Based on previous observations from different imaging modalities, we hypothesized alteration of CBF in the frontal cortex, thalamus, brainstem, and regions of the default mode network (Byas-Smith et al., 2002; Gili et al., 2013).

## Methods

Cardiff University’s School of Medicine Ethics Committee reviewed and approved the study. Fifteen right-handed, healthy, male volunteers (mean age 26 years; range 20–41 years) participated in this study after giving informed consent. They were recruited following a detailed screening procedure. Medical screening was performed to ensure that all subjects were in good physical and mental health and not on any medications (American Society of Anesthesiologists grade 1). Any volunteer with complaints of regular heartburn or hiatus hernia, known or suspected allergies to propofol (or its constituents), who was a regular smoker, or who snored frequently or excessively, or who had a potentially “difficult airway” was excluded. Volunteers were instructed to follow standard pre-anesthetic fasting guidelines. They avoided food for 6 h and any fluids for 2 h before the experiments. Following the experiments, they were monitored until they recovered from the effects of sedation and were discharged with safety advice after they fulfilled all day-case anesthesia discharge criteria. All participants underwent two fMRI scans within the same session, the first before and the second during intravenous propofol administration while remaining at rest. No behavioral task was presented apart from asking volunteers to remain still with their eyes closed and not to fall asleep.

### Drug Administration

Propofol (Propofol-Lipuro 1%, Braun Ltd.) was administered using an Asena-PK infusion pump (Alaris Medical, CareFusion Ltd.) using a target-controlled infusion based on the Marsh pharmacokinetic model (Marsh et al., 1991). Infusion was started targeting an effect-site concentration of 0.6 μg ml^−1^. Once the target was reached, 2 min were given for further equilibration. Drug infusion was increased in 0.2 μg ml^−1^ increments until the desired level of sedation was achieved. Sedation level was assessed by an anesthetist, blinded to the level of propofol being administered, using the modified Observer’s assessment of alertness/sedation (OAA/S) (Chernik et al., 1990). The sedation endpoint was an OAA/S level of 4 (slurred speech with lethargic response to verbal commands). The average targeted propofol plasma concentration was 1.2 (SD 0.2) μg ml^−1^. All subjects were monitored throughout the experiments by two qualified anesthetists (Table 1). Heart rate, non-invasive blood pressure, oxygen saturation, and concentrations of expired (end-tidal) carbon dioxide were monitored using aVeris MR Vital Signs monitoring system (MEDRAD Radiology).

**Table 1:** Physiological data.

	HR (bpm)	SBP (mmHg)	DBP (mmHg)	MAP (mmHg)	SpO_2_(%)
*Awake*	56 (7)	123 (10)	71 (8)	95 (8)	98 (1)
*Sedated*	55 (7)	119 (9)	70 (8)	92 (7)	98 (1)

*Mean (SD) across subjects of physiological recordings measured before and during sedation. Paired t-tests revealed no significant differences between awake and sedated states; SD, standard deviation; HR, heart rate; SBP, systolic blood pressure; DBP, diastolic blood pressure; MAP, mean arterial pressure; SpO_2_, oxygen saturation*.

### Magnetic Resonance Imaging

MRI data were collected at three T (General Electric, HDx) using an eight-channel receive-only head coil. CBF was estimated using single-shot, proximal inversion with a control for off-resonance effects – quantitative imaging of perfusion using single subtraction II (PICORE-QUIPSS II) (Wong et al., 1998). Imaging parameters were: TR/TE = 2,200 ms/19.8 ms; TI = 1,500 ms; field of view, 24 cm × 24 cm; 12 slices, 7 mm thick, with a 1-mm gap between slices; matrix, 64 × 64. Each scan included 130 repetitions (65 pairs of tag-control images) over 4:46 min. With the same slice prescription, calibration scans were acquired to provide an estimate of M_0_ (fully relaxed blood water magnetization). T1-weighted whole-brain structural scan was also acquired (1 mm × 1 mm × 1 mm voxels). Our previous work has demonstrated the robustness and repeatability of this sequence in a within-session design of experiment such as presented here (Murphy et al., 2011).

### Preprocessing

ASL data were pre-processed as follows: correction for head motion and removal of non-brain voxels were performed using FSL: FMRIB’s Software Library, www.fmrib.ox.ac.uk/fsl. Head motion correction was performed using MCFLIRT employing the same reference volume for both tag and control images. CBF data were processed using surround subtraction of the ASL tag and control images (Liu and Wong, 2005). CBF was calculated using a standard single-compartment model (Buxton et al., 1998). Data were then transformed first to individual subjects’ structural space using FLIRT (FMRIB’s Linear Registration Tool) and then to a standard space (Montreal Neurological Institute MNI152 standard map) using Advanced Normalization Tools (ANTS; Penn Image Computing & Science Lab, http://www.picsl.upenn.edu/ANTS/). The transformation was first applied to the individual subjects’ T1 image, and then the resulting warp vectors were applied to the CBF images.

### Analysis

To examine the influence of propofol sedation, permutation-based nonparametric within-subject paired *t-*tests (FSL randomize) were performed to identify areas where CBF varied significantly with the neurophysiological state (*awake* vs. *sedated*). The results were subject to threshold-free cluster enhancement and family-wise error (FWE) corrected for multiple comparisons by permutation testing using a significance level of *p* < 0.05.

## Results

There were no significant differences in systemic hemodynamics or oxygen saturation between the *awake* and *sedated* groups (Table 1). We compared head motion (relative volume to volume mean displacement) between awake and sedated groups, but there was no significant difference (two-tailed, paired *t*-test; *p* = 0.39).

**Figure 1:**
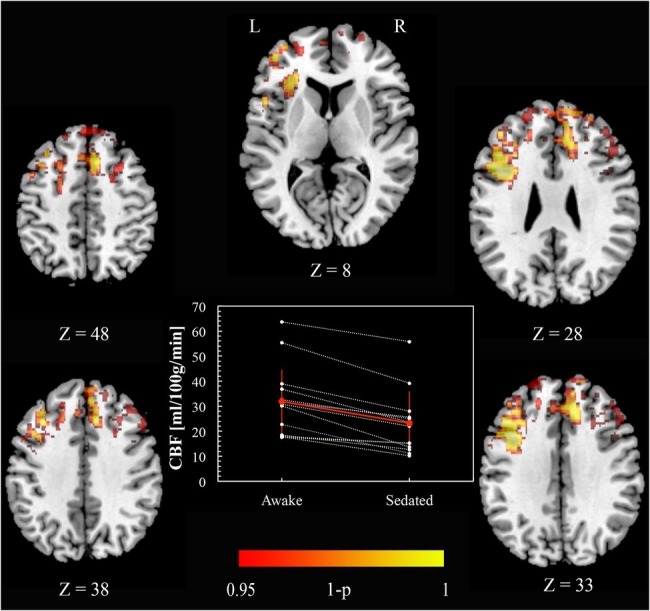
Cerebral blood flow changes induced by propofol mild sedation. A paired *t-*test was calculated for the decrease (*awake* > *sedated*) of the cerebral blood flow. We report clusters that survived correction for multiple comparisons across space, *via* permutation testing, with FWE correction at *p* < 0.05. The axial coordinates are reported in MNI space (mm). The central plot shows quantitative cerebral blood flow calculated within the region resulting from the paired *t*-test contrast (*awake* > *sedated*, *p* < 0.05 FWE corr.) across subjects. The red values represent mean and standard deviation in the two conditions.

Figure 1 shows CBF *awake* > *sedated* (paired *t* test), demonstrating frontal regions indicating a decrease in perfusion with sedation. Specifically, these areas were Paracingulate cortex [bilaterally (6, 34, 33) and (–4, 34, 33) MNI space coordinates], left and right Broca’s areas [(–38, 10, 28) and (42, 10, 28) MNI space, respectively], left Insula [(–32, 24, 8), MNI space], Premotor cortex [bilaterally (–4, 20, 48) and (8, 16, 48) MNI space], and right Superior Frontal gyrus [(4, 48, 38) MNI space]. No significant changes were found in the opposite contrast *awake* < *sedated*.

**Figure 2:**
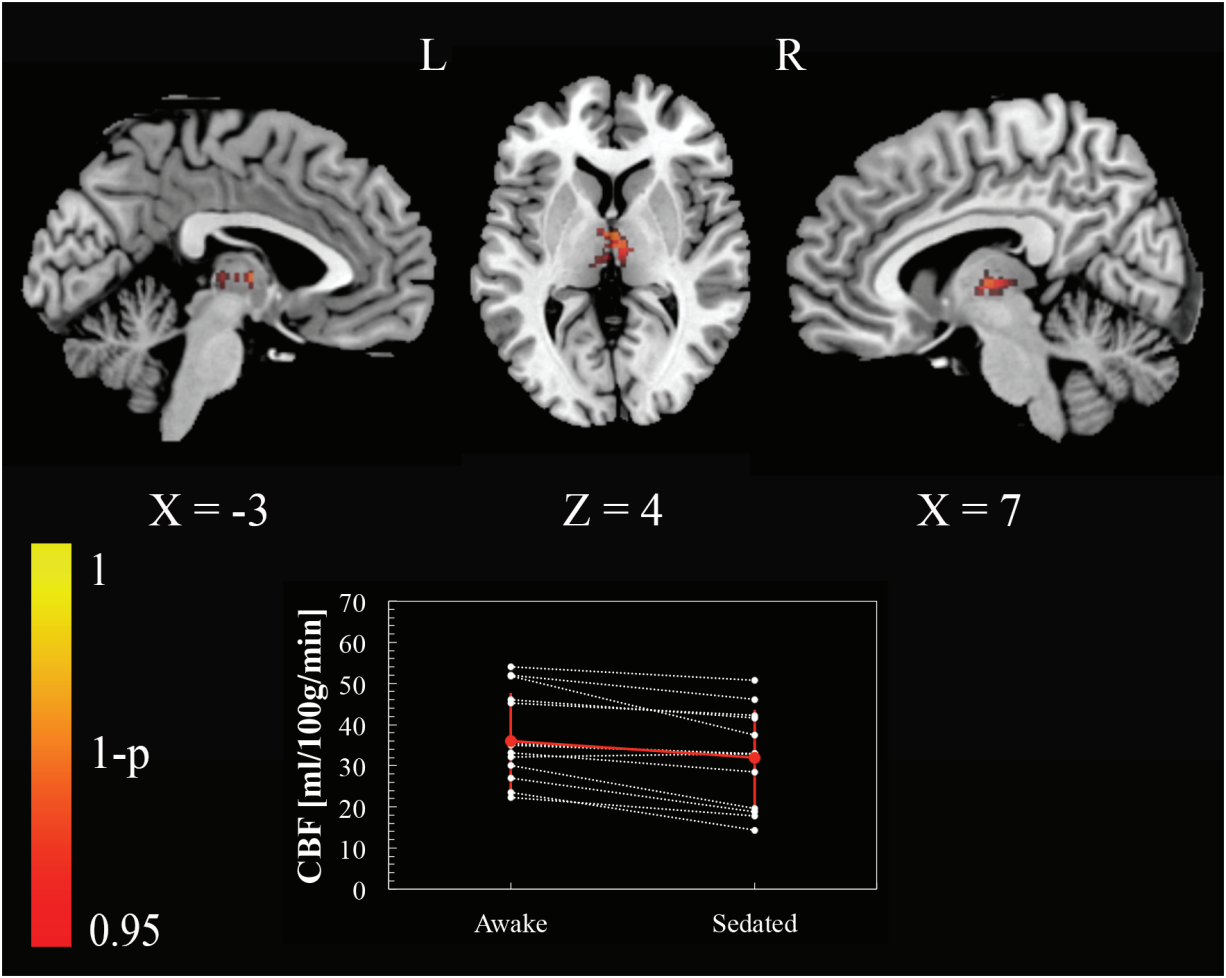
Cerebral blood flow changes induced by propofol mild sedation. A paired *t-*test was calculated for the decrease (*awake* > *sedated*) of the cerebral blood flow. We report clusters that survived small volume correction for multiple comparisons across space, *via* permutation testing, with FWE correction at *p* < 0.05. Coordinates are reported in MNI space (mm). The central plot shows quantitative cerebral blood flow calculated within the portion of thalamus that showed significant decrease in the paired *t*-test across subjects. The red values represent mean and standard deviation in the two conditions.

To test our regional hypotheses concerning areas commonly reported in studies of arousal, we calculated the paired *t*-test with a small volume FWE correction (*p* < 0.05), within thalamus, brainstem, and a set of regions (ventromedial prefrontal cortex, posterior cingulate cortex, left and right inferior parietal lobule) included in the default mode network (DMN) (Buckner et al., 2008). Only the thalamus bilaterally [(−3, −13, 4) and (7, −13, 4) MNI space coordinates] showed a reduction of perfusion passing from the awake condition to the sedated one (Figure 2).

## Discussion

In this experiment, we have used ASL-fMRI to demonstrate that mild propofol sedation is associated with a reduction in CBF in some of the key cortical (frontal lobe) and subcortical (thalamus) areas involved in modulating consciousness while the blood flow in the brainstem and the DMN remains broadly unaffected.

Previous quantitative studies of CBF changes have focused on anesthetic-induced unconsciousness, with only a few looking at mild sedation. In this study, we anticipated diminished regional perfusion of the frontal brain regions and thalamus based on previous anesthetic literature and the established role of these regions in attention, cognition, working memory, and consciousness. Mild sedation is characterized by alterations in attention, cognition, and memory. PET-based studies have demonstrated reduction in CBF of the frontal brain regions during both pharmacological sedation and anesthesia. Veselis et al. (2004) found a reduction in CBF in the right sided anterior brain (inferior frontal gyrus, insula, and superior temporal gyrus) with propofol doses similar to those in our study. Sun et al. (2008) also reported a reduction in frontal lobe metabolism by about 10 ± 3% and temporal lobe metabolism by 13 ± 2% during light propofol sedation but, simultaneously, a greater reduction in activity of the occipital lobe, an effect not found in our study. Occipital lobe has a high density of GABA receptors (similar to the frontal lobe), but its deactivation with sedation has not been universally reported. In our study, the subjects were instructed to keep their eyes closed, which may have contributed to a reduced baseline activation of the occipital lobe to restrict further significant changes. Byas-Smith et al. (2002) using propofol sedation (at doses higher than those in our study) showed a reduction in CBF of the middle and inferior frontal gyrus and additionally in the parietal lobe, posterior cingulate cortex, and also the thalamus.

We found reduced CBF, bilaterally, in the premotor cortex, paracingulate cortex, and Broca’s areas and right superior frontal gyrus. Prefrontal cortex plays an important role in attention and working memory. Dorsolateral prefrontal cortex is also functionally connected to parietal cortical regions forming the lateral frontoparietal attentional functional network, which becomes activated during executive tasks. Loss of frontoparietal feedback within these networks is associated with anesthetic-induced unconsciousness (Lee et al., 2009). Premotor cortex influences motor activity through its extensive connections with the primary motor cortex and also through the corticospinal and corticobulbar pathways. Anterior cingulate cortex plays an important role in cognitive processing, anticipation of incoming stimuli, attention and preparing and executing motor activity, both self-directed and in response to a verbal command. We have previously reported an increased connectivity of left premotor cortex and right ACC with the brainstem while their connectivity with thalamus was reduced with mild propofol sedation (Gili et al., 2013). Reduced perfusion of bilateral Broca’s areas (inferior frontal regions) was another expected finding as these are involved in language processing and perception; “slurred speech” being the hallmark of mild sedation (differentiating “no sedation” with “mild sedation” on the objective assessment of alertness/sedation scale).

We had hypothesized a reduction in CBF of the thalamus, brainstem, and regions involved in the DMN. Although we did not find significant CBF changes in the whole brain analysis in those areas, a small volume FWE corrected analysis, driven by our *a priori* regional hypothesis, revealed a reduction of thalamic perfusion. Most neuroimaging experiments investigating anesthesia have pointed toward a thalamic suppression along with a disruption of thalamocortical functional connectivity. At sedative concentrations of propofol, Byas-Smith et al. (2002) showed a dose-related reduction in regional perfusion of thalamus. Bonhomme et al. (2001) also showed a dose-related reduction in BOLD response activity of the thalamus to a sensory stimulus with increasing doses of sedation. At deeper levels of propofol sedation, thalamic perfusion is reduced (Qiu et al., 2017). It is therefore likely that the small doses of propofol required to produce mild sedation in our study affected the activity of brain networks in two steps: first a substantial reduction of blood perfusion in frontal areas as a consequence of local reduced metabolism associated with a small reduction of perfusion in the thalamus, followed by a progressive metabolic decrease, with increasing doses that leads to a gradual functional suppression of key node areas and their mutual interaction, resulting in anesthetic-induced unconsciousness.

We did not find any changes in regions involved in the DMN (including posterior cingulate, bilateral inferior parietal and medial prefrontal cortices). DMN functional connectivity has previously been shown to be preserved or partially maintained during propofol sedation (Boveroux et al., 2010; Stamatakis et al., 2010). Changes in functional connectivity of a set of areas may occur without any change in their mean activity levels. Functional connectivity between two regions is mainly related to the synchrony of the BOLD signal slow oscillations, rather than its magnitude of change. Similar to thalamic perfusion, CBF in the DMN decreased with increasing doses of propofol (Qiu et al., 2017) suggesting a step-wise, dose-related effect. We had also predicted changes in brainstem perfusion based on the findings of our previous work (Gili et al., 2013). There were no changes in brainstem perfusion changes observed. The low spatial resolution and limited coverage of the brainstem could be a factor in this finding. Qiu et al. (2017) also did not find brainstem perfusion changes with deeper levels of propofol sedation, suggesting that brainstem perfusion changes probably occur closer to anesthetic doses.

One of the challenges in deducing neural effects from cerebral blood flow changes is the potential confounding effect due to alterations of neurovascular coupling induced by physiological changes in blood oxygenation, CO_2_ concentration, and blood pressure, both as a consequence of drug effect on physiological variables and also the potential drug effect on neurovascular coupling *per se*. Propofol at sedative doses has been shown not to alter neurovascular coupling (Veselis et al., 2005).

The fixed order of awake state followed by the sedated state could be considered a limitation of the study design. However, within-session reproducibility of ASL measurements has previously been shown to be good, and better than between sessions, improving the power to detect drug effects with a within-session design (Murphy et al., 2011). Future study designs exploiting simultaneous BOLD and ASL to study coupling between changes in BOLD-CBF may also be useful in studying effects of sedation through alterations in oxygen metabolism where drug effects on neurovascular coupling render difficult interpretations of BOLD or CBF signals alone (Merola et al., 2017; Chiacchiaretta et al., 2018).

To conclude, we report the use of ASL to investigate the hemodynamic changes induced by mild propofol sedation. We have shown that the level of propofol sedation considered is associated with a reduction of CBF in key frontal areas (including bilateral premotor cortex, bilateral paracingulate cortex, bilateral Broca’s areas and right superior frontal gyrus) and in the thalamus, while the CBF in the brainstem and regions of the DMN was not significantly affected during mild sedation.

## Data Availability Statement

All datasets generated for this study are included in the article.

## Ethics Statement

The studies involving human participants were reviewed and approved by School of Medicine Research Ethics Committee, Cardiff University. The patients/participants provided their written informed consent to participate in this study.

## Author Contributions

NS contributed to study design, data acquisition, data analysis, data interpretation, manuscript preparation, and final approval. TG and AD contributed to study design, data acquisition, data analysis, data interpretation, critical revision of manuscript, and final approval. DH contributed to data acquisition, critical revision of manuscript, and final approval. JH contributed to study design, data interpretation, critical revision of manuscript, and final approval. RW contributed to study design, data analysis, data interpretation, critical revision of manuscript, and final approval.

### Conflict of Interest

The authors declare that the research was conducted in the absence of any commercial or financial relationships that could be construed as a potential conflict of interest.
